# SDHA related tumorigenesis: a new case series and literature review for variant interpretation and pathogenicity

**DOI:** 10.1002/mgg3.279

**Published:** 2017-03-02

**Authors:** Ruth T. Casey, David B. Ascher, Eleanor Rattenberry, Louise Izatt, Katrina A. Andrews, Helen L. Simpson, Benjamen Challis, Soo‐Mi Park, Venkata R. Bulusu, Fiona Lalloo, Douglas E. V. Pires, Hannah West, Graeme R. Clark, Philip S. Smith, James Whitworth, Thomas G. Papathomas, Phillipe Taniere, Rosina Savisaar, Laurence D. Hurst, Emma R. Woodward, Eamonn R. Maher

**Affiliations:** ^1^ Department of Medical GeneticsUniversity of Cambridge and NIHR Cambridge Biomedical Research CentreCambridgeCB2 2QQUK; ^2^ Department of EndocrinologyUniversity of Cambridge and NIHR Cambridge Biomedical Research CentreAddenbrooke's HospitalCambridgeCB2 2QQUK; ^3^ Department of BiochemistryUniversity of CambridgeSanger Building, 80 Tennis Court RoadCambridgeCB2 1GAUK; ^4^ Department of BiochemistryBio21 InstituteUniversity of MelbourneMelbourneVictoria3010Australia; ^5^ West Midlands Region Genetics ServiceBirmingham Women's HospitalBirminghamUK; ^6^ Department of Medical GeneticsGuy's HospitalLondonUK; ^7^ Oncology CentreCambridge University HospitalsCambridgeCB2 2QQUK; ^8^ Manchester Centre for Genomic MedicineSt Mary's HospitalCentral Manchester University Hospitals NHS Foundation TrustManchester Academic Health Science CentreManchesterUK; ^9^ Centro de Pesquisas René RachouFundação Oswaldo CruzBelo Horizonte30190‐002Brazil; ^10^ Department of HistopathologyKing's College HospitalLondonUK; ^11^ Histopathology and Cellular PathologyUniversity Hospitals Birmingham NHS Foundation TrustQueen Elizabeth HospitalBirminghamUK; ^12^ The Milner Centre for EvolutionDepartment of Biology and BiochemistryUniversity of BathBathBA2 7AYUK

**Keywords:** Pathogenesis, SDHA, variant

## Abstract

**Purpose:**

To evaluate the role of germline *SDHA* mutation analysis by (1) comprehensive literature review, (2) description of novel germline *SDHA* mutations and (3) in silico structural prediction analysis of missense substitutions in SDHA.

**Patients and methods:**

A systematic literature review and a retrospective review of the molecular and clinical features of patients identified with putative germline variants in UK molecular genetic laboratories was performed. To evaluate the molecular consequences of *SDHA* missense variants, a novel model of the SDHA/B/C/D complex was generated and the structural effects of missense substitutions identified in the literature, our UK novel cohort and a further 32 “control missense variants” were predicted by the mCSM computational platform. These structural predictions were correlated with the results of tumor studies and other bioinformatic predictions.

**Results:**

Literature review revealed reports of 17 different germline *SDHA* variants in 47 affected individuals from 45 kindreds. A further 10 different variants in 15 previously unreported cases (seven novel variants in eight patients) were added from our UK series. In silico structural prediction studies of 11 candidate missense germline mutations suggested that most (63.7%) would destabilize the SDHA protomer, and that most (78.1%) rare *SDHA* missense variants present in a control data set (ESP6500) were also associated with impaired protein stability.

**Conclusion:**

The clinical spectrum of *SDHA*‐associated neoplasia differs from that of germline mutations in other SDH‐subunits. The interpretation of the significance of novel *SDHA* missense substitutions is challenging. We recommend that multiple investigations (e.g. tumor studies, metabolomic profiling) should be performed to aid classification of rare missense variants before genetic testing results are used to influence clinical management.

## Introduction

Phaeochromocytoma (PCC) is a catecholamine secreting tumor arising from chromaffin tissue in the adrenal medulla. Similar tumors arising from sympathetic ganglion cells outside the adrenal are termed a paraganglioma (PGL) and are to be distinguished from head and neck paragangliomas (HNPGL), which are, in general, nonfunctional tumors arising from parasympathetic ganglia (Boulpaep et al. 2003). PCC/PGL are the most often inherited neuroendocrine tumors with approximately 40% of all cases harboring a genetic mutation in one of at least 13 genes (*NF1, RET, VHL, SDHB, SDHC, SDHD, SDHA, SDHAF2, MAX, TMEM127, HIF2A, MDH2*) (Yang et al. 2015; Lorenzo et al. 2013; Burnichon et al. 2010; Qin et al. 2010; Hao et al. 2009; Astuti et al. 2001a, 2001b; Baysal et al. 2000; Niemann and Müller 2000). In nonsyndromic cases of PCC/PGL, germline mutations are most frequently identified in *SDHB* and *SDHD* (Jafri et al. 2013; Neumann et al. 2004; Gimenez‐Roqueplo et al. 2003; Astuti et al. 2001a,b; Baysal et al. 2000), which encode the B and D subunits of the succinate dehydrogenase (SDH) complex (type II mitochondrial complex) which is an integral component of the citric acid cycle (Oyedotun and Lemire 2004). SDH facilitates the conversion of succinate to fumarate ensuring cellular metabolism of lipids, glucose and amino acids, and feeds into the mitochondrial respiratory chain to generate cellular energy (Oyedotun and Lemire 2004). Mutations in *SDHB* and *SDHD* have, in addition to PCC/PGL, also been associated with predisposition to HNPGL, renal cell carcinoma (RCC), gastrointestinal stromal tumors (GIST), and pituitary adenomas (PA) (Pantaleo et al. 2013; Xekouki and Stratakis 2012; Ricketts et al. 2010; Astuti et al. 2001a,b; Baysal et al. 2000).

The SDHD protein, together with SDHC, anchors the SDH complex to the inner mitochondrial wall and binds to SDHB, which in turn binds to SDHA (the catalytic subunit of the complex) (Oyedotun and Lemire 2004). Alhough mutations in *SDHB, SDHC,* and *SDHD* were associated with inherited PCC/PGL/HNPGL and mutations in *SDHA* were associated with autosomal recessive metabolic encephalopathy syndrome (Leigh syndrome) about 15 years ago (Horvath et al. 2006), the association of pathogenic germline *SDHA* mutations with inherited PCC was described only 5 years ago (Burnichon et al. 2012). In order to better characterize the genetic and clinical features of germline *SDHA* mutations, we undertook a literature review, and analyzed the clinical and molecular features of 15 new cases that found to have a germline *SDHA* variant via diagnostic testing and studied, computationally, the effect of novel and previously reported *SDHA* missense variants on SDHA structure. In addition, we assessed whether mutations might be predicted to disrupt splicing (Di Gaicomo et al. 2013; Woolfe et al. 2010; Wu and Hurst 2016; Pagani et al. 2005; Soukarieh et al. 2016), either by disrupting splice sites or by affecting exonic splice enhancers (Ke et al. 2011; Caceres and Hurst 2013) or silencers (Ke at al. 2011).

## Material and Methods

### Case series

Details of rare potentially pathogenic germline *SDHA* (OMIM: 600857, reference sequence: NG_012339.1) variants detected at UK NHS molecular diagnostic laboratories were obtained from those undertaking genetic testing. Referral data were collated on a standardized proforma and included: gender, age at presentation, method of presentation (sporadic vs. familial), location of tumor, presence of bilateral/multifocal disease, and evidence of malignancy. Malignancy was defined as the presence of distant or local regional metastasis. Patients gave written informed consent to a research ethics committee approved research study and/or data was collected as part of a molecular genetics service evaluation study.

### Literature review

A *SDHA* mutation search in association with PCC/PGL, GIST, RCC, PA, Leigh syndrome, and optic atrophy was performed. This search was performed using the Human Gene Mutation Database (www.hgmd.cf.ac.uk), the Leiden Open Variation Database (http://www.lovd.nl/3.0/home), and publications indexed in PubMed (http://www.ncbi.nlm.nih.gov/pubmed) up to May 2016. The following search terms were used: *SDHA* mutation in combination with the terms: phaeochromocytoma, paraganglioma, GIST, pituitary adenoma, renal cell carcinoma, and the conditions Leigh syndrome and optic atrophy. Both germline and somatic variants were included in the search and the results were subcategorised for germline versus somatic variants identified.

### Assessment of variant pathogenicity

In cases where the detected *SDHA* variant identified was novel and suspected to be causative in the disease phenotype, classification of variant pathogenicity was performed based on the recently published classification system by the American College of Genomic Medicine (ACMG) (Richards et al. 2015). This system categorizes variants as pathogenic or benign. If a variant does not meet the criteria for either a pathogenic or a benign variant, the recommendation is that this variant be defaulted to a variant of uncertain significance. Within the pathogenic category, variants can be further subclassified as either; pathogenic or likely pathogenic. Similarly within the benign category, sub classification includes benign or likely benign (Richards et al. 2015).

The criteria used to classify a variant included; review of the disease phenotype, the use of the predictive tools SIFT and Poly‐Phen2 and when available, review of functional tumor studies (including immunohistochemical staining (IHC) of the SDHB/SDHA protein and loss of heterozygosity studies (LOH)). The presence of the disease allele in a healthy control population was also confirmed by searching the EXAC database (http://exac.broadinstitute.org/). Variants identified in the literature which, were not considered to be disease causing by the authors were excluded.

Information from computational predictive tools above and functional studies when available was combined with in silico predicted changes in protein stability and protein‐protein affinity upon mutation for 18 missense variants identified. This information was compiled and variants were classified as per the ACMG recommendations. An online genetic variation tool predictor (http://medschool.umaryland.edu/Genetic Variant_Interpretation_Tool1.html) based on ACMG guidelines was used to tabulate the evidence for the 18 missense variants (see Table 2).

### Modeling of the SDHA/B/C/D complex and prediction of the effects of missense substitutions

A molecular model of SDHA was generated using Modeller and Macro Model (Schrodinger, New York, NY) using the X‐ray crystal structures of Succinate dehydrogenase flavoprotein subunit from the Avian respiratory complex ii (92% sequence identity; PDB ID: 1YQ4) (Huang et al. 2006) and the Flavoprotein subunit of Complex ii from Ascaris suum (72% sequence identity; PDB ID: 3VR8) (Shimizu et al. 2012). The models were then minimized using the MMF94s forcefield in Sybyl‐X 2.1.1 (Certara L.P, St Louis, MO, USA), with the final structure having more than 95% of residues in the allowed region of a Ramachandran plot. The FAD cofactor and Succinate substrate were docked into the models using Glide (Schrodinger), and the position of the ligands in available crystal structures used to guide placement. The quality of the models was confirmed with Verify3D (data not shown). Model structures were examined using Pymol. The model of the succinate complex was built using our previously reported models of SDHB and SDHD, with the X‐ray crystal structure of the Avian respiratory complex ii (PDB ID: 1YQ4) (Huang et al. 2006) was used to guide protein docking.

The structural consequences of all the identified novel and previously identified *SDHA* missense variants were analyzed to account for all the potential effects of the mutations (Pires et al. 2016). The effects of the mutations upon the stability of SDHA were predicted using DUET (Pires et al. 2014a,b), an integrated computational approach that optimizes the prediction of two complementary methods (mCSM‐Stability and SDM). The effect of the mutations upon the protein–protein binding affinity of SDHA to form the succinate complex were predicted using mCSM‐PPI (Pires et al. 2014a; Pires and Ascher 2016). The effect of the mutations upon the binding affinity of SDHA for the cofactor, FAD, and substrate, succinate, were predicted using mCSM‐Lig (Pires et al. 2015, 2016). These computational approaches represent the wild‐type residues structural and chemical environment of a residue as a graph‐based signature in order to determine the change upon mutation in Gibb's free energy of stability or binding. To compare the in silico predictions for germline *SDHA* missense mutations detected in patients with those not ascertained via diagnostic testing, we identified 24 rare (frequency <0.01%) germline *SDHA* missense variants present in the ESP6500 cohort from Exome Variant Server (http://evs.gs.washington.edu) and correlated the effect of these missense variants on protein stability, complex formation, and ligand binding using our in silico prediction approaches. In addition, eight presumed missense somatic *SDHA* variants detected in SDH‐related tumor types (seven renal cell carcinomas and one phaeochromocytoma) from the cBioPortal for cancer genomics (http://www.cbioportal.org), were evaluated.

### Modeling of mammalian alignment to detect domains of purifying selection using SDHA transcript

Mammalian alignment from the 100 vertebrate genomes alignment for NM_004168.2 was downloaded via Table Browser at UCSC https://genome.ucsc.edu/cgi-bin/hgTables. We calculated mean Ks (human to comparator) and % gap in the alignment, and selected sequences with <5% gap and <0.45 Ks (to avoid saturation problem) but >0.1 Ks (to ensure adequate information). Baboon sequence was eliminated owing to an in‐frame stop. These filters resulted in two human‐primate comparators, these being Human‐marmoset (*Callithrix jacchus, calJac3 assembly)* and human‐ bushbaby *(Otelemur garnetti otoGar3 assembly)*. The two alignments were passed by package SLIDERKK.tcl (available form LDH) to calculate Ka/Ks ratios in 108‐bp windows. We employed Li93 as the method of Ka/Ks calculation. We reviewed the variants identified in this study to determine which variants plotted to domains of strong purifying selection on this mammalian alignment.

### Predicting the effects of the variants on splice regulatory information

See Appendix S7.

### Statistical analysis

Statistical tests were performed using SPSS. Student's *t*‐test was used to compare continuous variables and Mann–Whitney or an unpaired *t*‐test to compare nonnormally distributed data when sample numbers were small. Summary statistics included means and standard deviations for continuous variables, and frequencies and percentages for categorical variables.

## Results

### UK *SDHA* germline mutation series

#### Molecular genetics

Fifteen previously unreported patients with ten different germline *SDHA* variants were identified (Table 1). Two mutations had been reported previously: the common nonsense mutation c.91C>T (p.Arg31*) was observed in five patients and a c.1753C>T (p.Arg585Trp) missense mutation in one patient. A novel truncating mutation in c.1468G>T (p.Glu490*) was identified in one patient with a metastatic GIST tumor. Four further novel candidate missense variants, one frameshift variant and one splice acceptor variant were detected in six kindreds (see Table 1) (a novel missense variant, c.923C>T (p.Thr308Met) in exon 8 of *SDHA* was detected in two apparently unrelated patients).

**Table 1:** Clinical phenotype of patients with variants in *SDHA* in novel UK cohort

Mutation	Sex	Age	Category	Single/multiple	Secretory	Malignant
c.91C>T (p.Arg31*)	M	56	HNPGL	Single	No	No
c.91C>T (p.Arg31*)	M	33	Abdominal PGL	Single	N/A	No
c.91C>T (p.Arg31*)	M	45	Abdominal PGL	Single	Yes	No
c.91C>T (p.Arg31*)	F	15	Adrenal PCC	Single	Yes	No
c.91C>T (p.Arg31*)	M	35	GIST	Single	No	Yes
c.133G>A (p.Ala45Thr)	M	36	Thoracic PGL	Single	No	No
c.136A>G (p.Lys46Glu)	F	12	Abdominal PGL	Single	Yes	No
c.923C>T (p.Thr308Met)	F	43	Thoracic PGL	Single	Yes	Yes
c.923C>T (p.Thr308Met)	M	52	HNPGL	Multiple	Yes	No
c.1273G>A (p.Val425Met)	M	62	PC and Paraspinal PGL.	Multiple	Yes	No
c.1338delA (p.His447Metfs*23)	F	48	HNPGL	Single	No	No
c.1468G>T (p.Glu490Ter)	M	32	GIST	Single	No	Yes
c.1753C>T (p.Arg585Trp)	F	34	PGL	Single	No	No
c.1765C>T (p.Arg589Trp)	F	42	GIST	Single	No	No
c.1909‐2A>G	F	31	GIST	Single	No	No

#### Clinical features

Four patients presented with a GIST and eleven patients presented with a PCC/PGL. The mean age of disease presentation was 37.1 years (SD 14.2) with a range of 12–65 years. None of the affected individuals had a family history of SDH‐related tumors. One proband with a truncating *SDHA* mutation (c.91C>T p.Arg31*) had a first degree relative tested after diagnosis who is an asymptomatic mutation carrier at age 72 years. One patient had died from another disease process at the time of this review. One patient with the c.923C>T (p.Thr308Met) missense variant was diagnosed with a malignant mediastinal paraganglioma at age 43 years and the second patient presented at a later age (52 years) with multiple bilateral HNPGL and a unilateral PCC. Further unreported variants included a missense variant in c.1273G>A (p.Val425Met) in a 62‐year‐ old gentleman presenting with a para‐spinal PGL and unilateral PC, two further missense mutations; c.133G>A (p.Ala45Thr) in exon 2 in a young male with a mediastinal PGL and c.136A>G (p.Lys46Glu) in exon 2, which was detected in a girl presenting of age 12 with a porta hepatis PGL. A novel truncating mutation [c.1468G>T (p.Glu490*)] was identified in a male patient, who presented aged 32 years with a GIST and later aged 36 and 38 years developed liver and lung metastases. The final two novel variants detected included a frameshift mutation (c.1338delA) in a 48‐year‐old female with a HNPGL and a splice mutation (c.1909‐2A>G) in a 31‐year‐old female with a GIST.

### Literature review of germline and somatic *SDHA* mutations

#### Germline SDHA mutations

Of 17 unique germline *SDHA* variants were identified in 47 individuals from 45 kindreds (Table S1). Three recurrent germline variants were identified: c.91C>T (p.Arg31*) nonsense variant in 22 kindreds (23 affected individuals) and two missense variants: c.1753C>T (p.Arg585Trp) (in two kindreds and two affected individuals) and c.1765C>T (p.Arg589Trp) (in four kindreds and four affected individuals). Details of clinical phenotype (Table S1) revealed that the most common association was with GIST tumors (mean age at diagnosis 33.4 years (SD+11.1), range 17–62 years) occurring in 31 of the 47 affected individuals. Five reported cases of metastatic GIST with *SDHA* germline variants were identified: two cases in association with a c.91C>T (p.Arg31*) (Pantaleo et al. 2011a,b; Italiano et al. 2012) and three further cases of metastatic GIST have been published in patients with the following mutations in *SDHA*: c.1151C>G (p.Ser384*) (Pantaleo et al. 2011a,b), c.1765C>T (p.Arg589Trp) (Wagner et al. 2013), and c.1534C>T (p.Arg512*) (Wagner et al. 2013) Reports of the PCC/PGL phenotype included eight PGL (abdominal or thorax), four HNPGL, and one PCC. There were two reports of malignant PCC/PGL in association with germline *SDHA* variants. One patient with a sympathetic bladder PGL and a c.91C>T (p.Arg31*) variant (Burnichon et al. 2012), and a second patient with a HNPGL and a c.1534C>T (p.Arg512*) variant (Papathomas et al. 2015). No case of multifocal PCC/PGL was identified. One report of nonfunctioning pituitary marcoadenoma and a germline *SDHA* variant was identified (Dwight et al. 2013a). Three recent case reports of renal cell carcinoma (RCC) in association with a *SDHA* variant have been published (Jiang et al. 2015; Ozluk et al. 2015; Yakirevich et al. 2015). One patient had a novel germline variant in c.2T>C (p.M1T) in the initiation codon of *SDHA* (Jiang et al. 2015) and was diagnosed with a renal cell chromophobe tumor and a multifocal GIST tumor. The two further reports were associated with somatic mutations and are described below.

An incomplete penetrance pattern with *SDHA* mutations is suggested by the sparse number of familial cases identified. Only two familial *SDHA* mutations were reported: two sisters with a c.91C>T (p.Arg31*) variant and GIST (Oudijk et al. 2013) and an additional family with a c.1873C>T (p.His625Tyr) variant, where the mother was the proband and had a HNPGL and her son had a nonfunctioning PA (Dwight et al. 2013a). The characteristics and population frequency of individual *SDHA* mutations described in the literature are described in Tables S1 and S2. The recurrent c.91C>T (p.Arg31*) nonsense mutation is recorded as occurring in 0.2 per 1000 individuals in the EXAC database (exac.broadinstitute.org/about) and all except one of the putative germline variants in our UK series and in the literature had a frequency of <1 per 1000 individuals in the EXAC dataset. However, a c.113A>T (p.Asp38Val) missense substitution described (Italiano et al. 2012) as a somatic mutation in a 26‐year‐old *female with* a metastatic GIST tumor with liver and peritoneal metastasis was present in 3.5% of individuals in the EXAC database.

A total of nine germline variants (three missense, six truncating) in *SDHA*, associated with either optic atrophy or Leigh syndrome were identified in the literature (Table S3). The only germline variant associated with both Leigh syndrome/optic atrophy and tumorigenesis including GIST and PCC/PGL was the c.91C>T (p.Arg31*) truncating variant.

#### Somatic SDHA mutations

Eleven cases of somatic candidate *SDHA* mutations were identified in the literature (Table S1): seven missense variants and four truncating. The associated tumor types included: GIST (*n* = 8), RCC (*n* = 2) and PA (*n* = 1). Two cases of RCC are associated with somatic SDHA variants (Ozluk et al. 2015; Yakirevich et al. 2015) and had histologic features, which were consistent with the histology typically associated with *SDHB* associated RCC (Ozluk et al. 2015; Yakirevich et al. 2015). One patient with a novel somatic 17 kbp *SDHA* homozygous deletion on chromosome 5p15, had malignant RCC (Yakirevich et al. 2015).

### In silico structural analysis of germline and somatic *SDHA* variants associated with tumorigenesis

Computational approaches were employed to assess the effects of mutations on protomer stability, complex formation and ligand binding to classify all identified *SDHA* missense variants associated with tumorigenesis in the literature and our unpublished cohort. A total of 18 putative missense mutations (11 germline and seven somatic) were analyzed. The data obtained from this in silico analysis were compiled with other predictive tools and a classification of these missense variants was made based on the ACMG recommendations (Richards et al. 2015) from the existing criteria available on each variant.

The mean DUET stability score was −0.52 kcal/mol (SD 0.936) for the 18 missense variants associated with tumorigenesis (mean −0.53 kcal/mol for 11 germline variants and −0.48 kcal/mol for seven somatic variants. The mean DUET score for missense variants (*n* = 3) reported in association with Leigh syndrome/optic atrophy was −1.15 kcal/mol.

The most destabilizing germline mutation predicted by DUET was −1.81 kcal/mol and associated with the missense variant c.1766G>A (p.Arg589Gln). This missense variant was detected in a single case of GIST in the literature. The second highest DUET score was associated with the germline missense variant c.1765C>T (p.Arg589Trp) which was identified in one patient in our study cohort with a GIST, and has been identified in the literature in one other patient with GIST and two patients with paragangliomas (see Table 3). Overall the most destabilizing mutation predicted by DUET was −3.1 kcal/mol and associated with a somatic mutation (c.1361C>A p.Ala454Glu) identified in a single case of GIST in the literature (see Table S1). Interestingly this variant was associated with loss of SDHB staining on immunohistochemistry but retained SDHA staining. This in silico prediction tool, predicted that the variant was highly destabilizing. It is in the FAD binding pocket and the mutation would abolish FAD binding and disrupt formation of the succinate complex.

The variant c.923C>T (p.Thr308Met) identified in two unrelated patients in our cohort with aggressive phenotypes (see Table 1) was predicted to mildly destabilize the protein protomer and part of substrate binding with a DUET score of −0.498 kcal/mol. No significant differences were detected between DUET scores of missense variants associated with GIST and with PCC/PGL (*P* = 0.2).

The in silico prediction tool predicted that 8/18 missense variants analyzed would have a mild or no effect on protein stability. Two of the eight variants were somatic variants; c.113A>T (p.Asp38Val) identified in a single GIST in the literature and the c.1334C>T (p.Ser445Leu) variant, also detected in a single GIST in the literature. The remaining six variants were germline and four of the six variants were identified in our novel UK cohort; c.133G>A (p.Ala45Thr), c.136A>G (p.Lys46Glu), c.923 C>T (p. Thr308Met), c. 1273G>A (p.Val425Met). A potential alternative mechanism for pathogenicity could be postulated for three N‐terminal missense substitutions: c.113A>T (p.Asp38Val), c.133G>A (p.Ala45Thr) and c.136A>G (p.Lys46Glu), which were predicted to affect the transit peptide and potentially alter protein localization (see Table 3). One variant, c.1690G>A (p.Glu564Lys), was predicted to destabilize complex formation by mCSM‐PPI (score of −0.951 kcal/mol).

A total of 8 somatic missense *SDHA* variants were identified from the cBioportal (http://www.cbioportal.org) in tumors associated with the *SDHA* disease phenotype. The mean DUET score in this group was −0.94 kcal/mol and 75% (6/8) of the missense variants were predicted to destabilize the protein or its ability to bind the substrate or form a complex (see Table S5). Three of these mutations were also predicted to affect complex formation (average mCSM‐PPI score of −1.025 kcal/mol).

### In silico structural analysis of germline *SDHA* variants in control dataset

If *SDHA* pathogenic variants are usually associated with a low penetrance phenotype it might be postulated that rare pathogenic variants might also be detected in the general population. We therefore analyzed 24 rare (<0.05%) missense variants reported in a control data set (EVS6500, http://evs.gs.washington.edu) but not present in patient cohorts (Table S4) for predicted effect on protein stability, protein–protein and protein–ligand affinity and found that most 75% (18/24) were predicted to have a destabilizing effect and 41.6% (10/24) were predicted to affect complex formation. The in silico predictions of DUET correlated with SIFT and Polyphen prediction tools for 58.3% (14/24) of the variants. Additionally, 75% (6/8) of the somatic missense variants identified in RCC and 1 PCC tumor described in the CBioportal database were predicted to destabilize the protein (7/8) were predicted to be deleterious by SIFT/Polyphen) (see Table S5).

### Tumor analysis in UK cohort

Two tumor specimens from our unpublished cohort were available for analysis. *SDHA* sequence analysis on a PGL from a patient with a c.1753 C>T variant (p.Arg585Trp) demonstrated partial loss of the wild‐type allele in the tumor DNA consistent with pathogenicity. Tumor tissue from a patients with a c.91C>T (p.Arg31*) confirmed the presence of the variant but no loss of the wild type allele was detected (data not shown).

### Classification of potential pathogenicity of germline *SDHA* variants associated with disease in our cohort and literature

Data from the in silico protein stability and affinity predictions was collated with data from computational predictive analyses and tumor studies in order to classify 18 identified missense variants as per the ACMG guidelines (Richards et al. 2015). 13/18 (72.2%) missense variants met the criteria for a pathogenic (or likely pathogenic) mutation (see Table 2). Five variants (29.5%) did not meet the criteria for a pathogenic mutation, and the supporting evidence was supportive of a likely benign variant for four variants and one variant was classified as a variant of uncertain significance (VUS) because of insufficient evidence to classify as benign or pathogenic.

**Table 2:** Classification of potential pathogenicity of *SDHA* missense variants identified in literature and novel UK cohort as per ACMG guidelines

Variant	Effect	Evidence
c.113A>T (p.Asp38Val)	Likely benign (II)	PP5, PP4, BP1, BP4, BS1
c.133G>A (p.Ala45Thr)	Likely benign (II)	PP4, BP1, BP4, PS3
c.136A>G (p.Lys46Glu)	Likely benign (II)	PP4, BP1, BP4
c.511C>T (p.Arg171Cys)	Likely pathogenic (III)	PS3, PP4, BP1, PP3
c.562C>T (p.Arg188Trp)	Likely pathogenic (III)	PS3, PP3, PP4, BP1
c.767C>T (p.Thr256Ile)	Likely pathogenic (III)	PS3, PP3, PP4, BP1
c.800C>T (p.Thr267Met)	Likely pathogenic (III)	PS3, PP3, PP4, BP1
c.923C>T (p.Thr308Met)	VUS ‐ not enough evidence	BP1, PP3
c.1255G>A (p.Gly419Arg)	Likely pathogenic (III)	PP4, PP3, PS3, BP1
c.1273G>A (p.Val425Met)	Likely benign (II)	BP1, PP4, BP4
c.1334C>T (p.Ser445Leu)	Likely pathogenic (III)	BP1, PP3, PS3, PP4
c.1361C>A (p.Ala454Glu)	Likely pathogenic (III)	PS3, PP3, PP4, PP5
c.1690G>A (p.Glu564Lys)	Likely pathogenic (III)	PS3, PP4, PP3, BP1
c.1753C>T (p.Arg585Trp)	Likely pathogenic (III)	PS3, PP3, PP4, BP1
c.1765C>T (p.Arg589Trp)	Likely pathogenic (III)	PS3, PP5, PP3, PP4, BP1
c.1766G>A (p.Arg589Gln)	Likely pathogenic (III)	PS3, PP4, PP5, PP3, BP1
c.1794G>C (p.Lys598Asn)	Likely pathogenic (III)	PS3, PP3, PP4, PP5, BP1
c.1873C>T (p.His625Tyr)	Likely pathogenic (III)	PS3, PP3, PP4, PP5, BP1

The five variants included four novel variants identified in our UK cohort and one variant identified from the literature. The first variant c.113A>T (p.Asp38Val), was a somatic variant and identified from the literature in a patient with a metastatic GIST. This variant was predicted to be benign by SIFT and Polyphen 2 prediction and is frequently seen in healthy controls (2.1%). It was predicted to cause potential disruption to the transit peptide on our in silico structural analysis and tumor analysis was reported to show loss of heterozygosity and loss of SDHA immunostaining (one limitation to this study was only three *SDHA* exons were sequenced and another undetected mutation might have been present *in cis*) (Italiano et al. 2012)^.^

**Figure 1:**
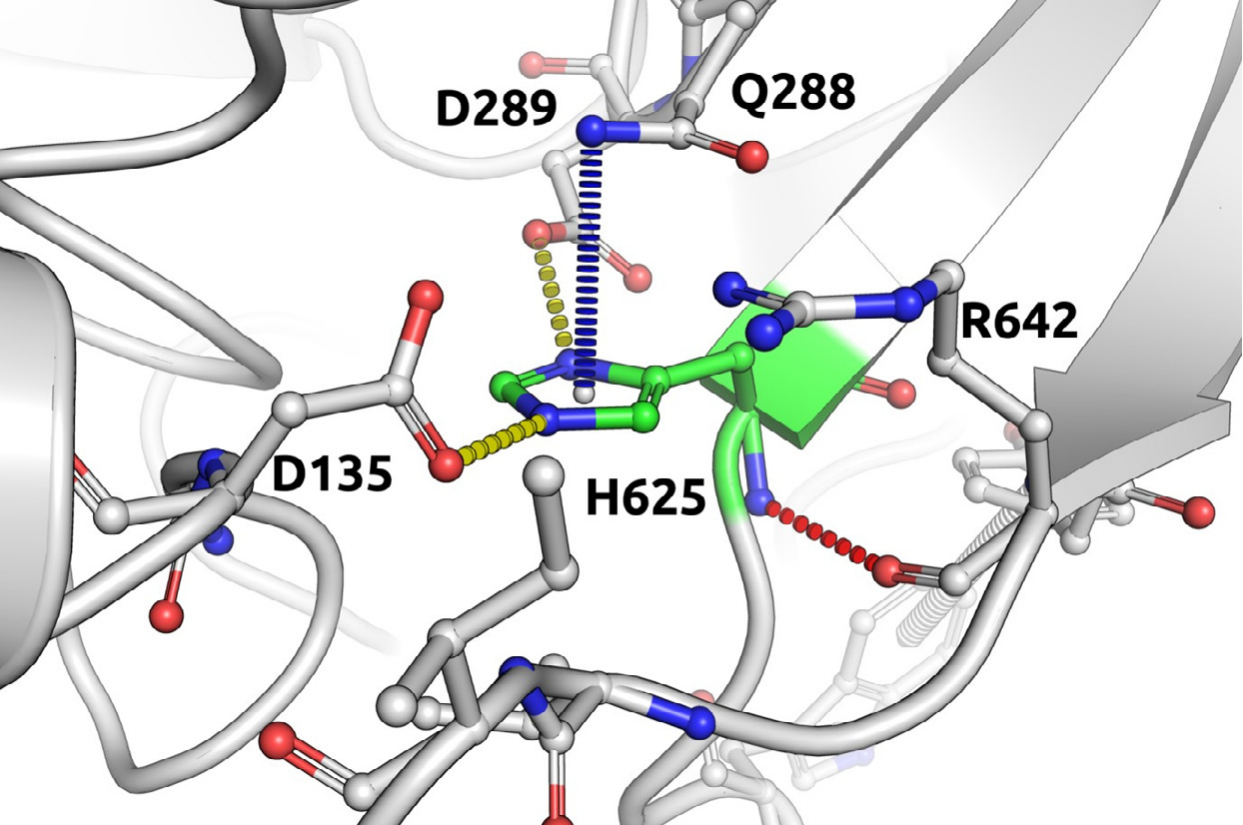
Molecular depiction of the effect on protein caused by c.1873C>T (p.His625Tyr) SDHA mutation.

Interestingly the variant c.1873C>T (p.His625Tyr), classified as likely pathogenic as per ACMG (Richards et al. 2015), was not shown to have any effect on protein stability in our in silico analysis (Table 3). This variant was identified in a patient with a PGL (proband) and her son who was diagnosed with a pituitary adenoma (Dwight et al. 2013a). No loss of the wild type allele was demonstrated in the pituitary adenoma but loss of SDHA and SDHB immunostaining was demonstrated in both tumor types. Taking a closer look at this mutation at a molecular level, however, reveals that His625 establishes an intricate network of polar interactions, including ionic interaction with Asp135 and Asp289, a donor–pi interaction with a Gln288 and a main‐chain to main‐chain hydrogen bond with Arg642 (depicted in Fig. 1). These would most likely be disrupted by the mutation to Tyr, destabilizing the protein.

**Table 3:** Structural Impact of 18 *SDHA* Missense substitutions on in silico protein models and correlation with other predictive tools

Nucleotide	Phenotype	DUET score (kcal/mol)	mCSM‐PPI score (kcal/mol)	Effect on protein	Effect on co‐factor	SIFT/Polyphen prediction	Heterozygous frequency per 1000 healthy population
c.113A>T (p.Asp38Val)	GIST	NA	NA	Transit peptide	No	Benign	21.7
c.133G>A (p.Ala45Thr)	Thoracic PGL	NA	NA	Near transit peptide	No	Benign	0.34
c.136A>G (p.Lys46Glu)	Abdominal PGL	NA	NA	Near transit peptide	No	Benign	0.24
c.511C>T (p.Arg171Cys)	GIST	−1.183	−0.592	Destabilizes protomer and complex	Yes	Damaging	Not described
c.562C>T (p.Arg188Trp)	GIST	−0.901	−0.235	Destabilizes protomer	Yes	N/A	Not described
c.767C>T (p.Thr256Ile)	GIST	−0.397	−0.397	Destabilizes protomer and complex	Yes	Probably damaging	Not described
c.800C>T (p.Thr267Met)	GIST	0.77	−0.287	Substrate binding pocket	Yes	Probably damaging	Not described
c.923C>T (p.Thr308Met)	HNPGL, Thoracic PGL	−0.498	−0.16	Mildly destabilizes protomer and part of substrate binding site	No	Benign	Not described
c.1255G>A (p.Gly419Arg)	GIST	−1.268	0	Destabilizes protomer	No	Probably damaging	Not described
c.1273G>A (p.Val425Met)	PGL	0.083	0	No effect	No	Probably damaging	0.02
c.1334C>T (p.Ser445Leu)	GIST	0.971	0	Stabilizes protomer	No	Probably damaging	Not described
c.1361C>A (p.Ala454Glu)	GIST	−3.1	−1.65	Destabilizes complex	Yes	Damaging	Not described
c.1690G>A (p.Glu564Lys)	GIST	0.263	−0.951	Destabilizes complex	Yes	Probably damaging	Not described
c.1753C>T (p.Arg585Trp)	PGL, PC	−1.09	0	Destabilizes protomer	No	Damaging	0.002
c.1765C>T (p.Arg589Trp)	GIST, PGL	−1.383	0	Destabilizes protomer	No	Damaging	Not described
c.1766G>A (p.Arg589Gln)	GIST	−1.81	0	Destabilizes protomer	No	Probably damaging	Not described
c.1794G>C (p.Lys598Asn)	GIST	0.301	0	No effect	No	N/A	0.016
c.1873C>T (p.His625Tyr)	1 PA, 1 HNPGL	0.059	0	No effect	No	N/A	Not described

The three remaining variants classified as likely benign and the variant classified as a VUS were identified in our novel cohort. One limiting factor to this classification was that tumor tissue was not available and so the variants could not be fully assessed. The first two variants c.133G>A (p.Ala45Thr) detected in a patient with a thoracic PGL and the c.136A>G (p.Lys46Glu), identified in a patient with an abdominal PGL, were consistently predicted as benign variants across different computational analysis tools and occurred at a frequency of up to 0.03% in healthy controls (see Table 1). These variants were predicted as having a potential effect on the transit peptide but the DUET, mCSM‐PPI and mCSM‐Lig scores could not be calculated and there was no effect on the cofactor.

**Figure 2:**
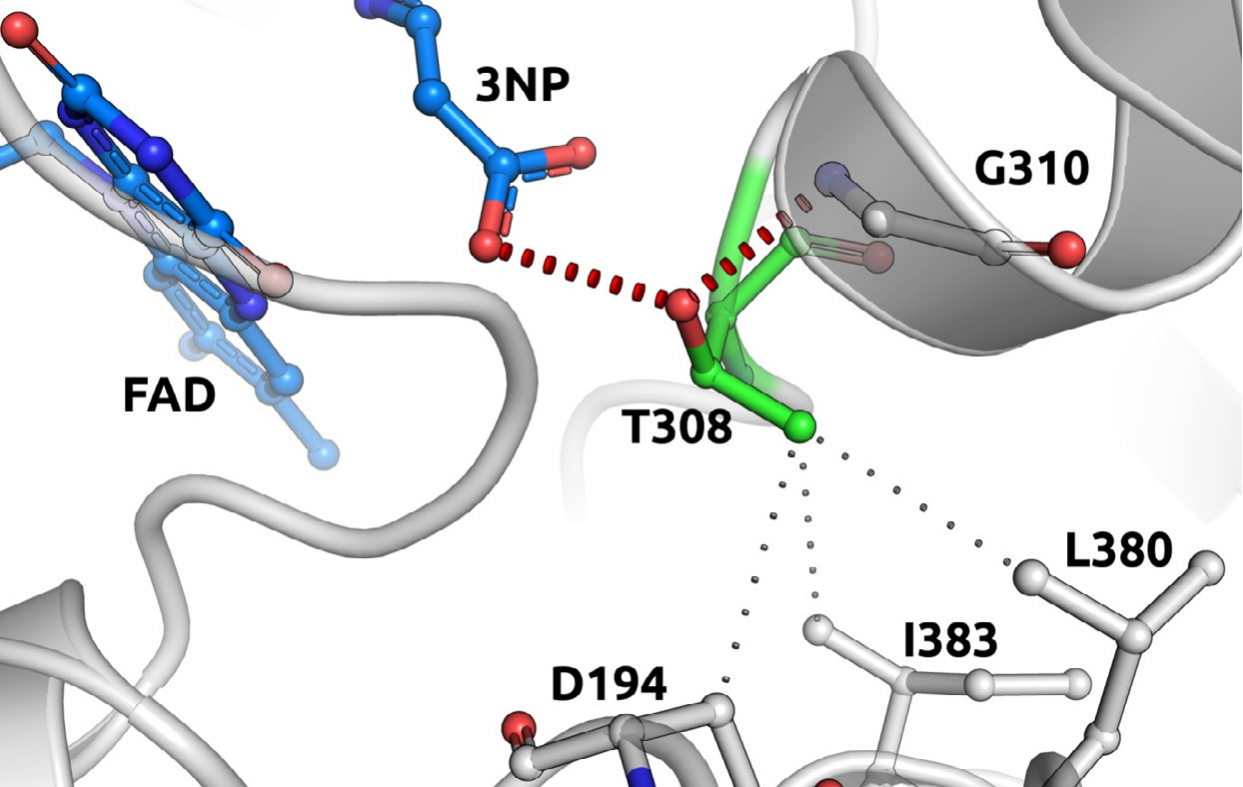
Molecular depiction of the effect on protein caused by c.923C>T (P.Thr308Met) SDHA mutation.

The remaining variants were suspected to be pathogenic. The first variant c.923C>T (p.Thr308Met), was detected in two patients who are not known to be related. This variant was associated with a malignant mediastinal PGL in one patient and multiple PGL and a PCC in a second patient. This variant was predicted to be benign by SIFT and PolyPhen but has not been identified in healthy controls and was also found to mildly destabilize the protomer and substrate binding site and therefore is likely to affect protein stability. Thr308 establishes, apart from hydrophobic interactions, hydrogen bonds that would be lost by the substitution to Methionine, which could also induce steric clashes (Fig. 2). Its proximity to the ligand FAD, could also imply a change in substrate binding as well. However due to insufficient evidence, the default classification of this variant was VUS. The final variant c.1273G>A (p.Val425Met), was detected in a patient with a spinal PGL and PCC. This variant was predicted to be pathogenic by SIFT and PolyPhen and is only present in .002% of healthy controls. However this variant was not found to impact on protein stability by our in silico prediction analysis. As functional studies were not performed on this variant the overall criteria for a likely pathogenic variant were not met and the classification was a likely benign variant as per ACMG (see Table 2).

### Prediction of splice disruption

Appendix S7.

### Variants plotted to domains of strong purifying selection on mammalian alignment

**Figure 3:**
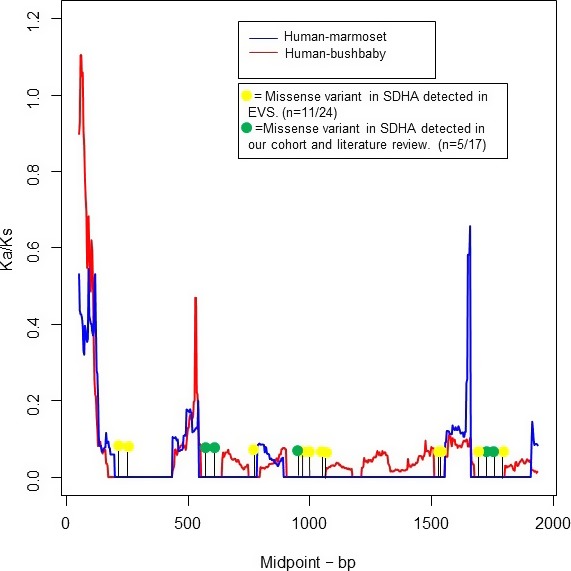
Modeling of mammalian alignment to detect domains of purifying selection using SDHA transcript.

A priori we might expect disease causing mutations to be more common in domains of high‐conservation within a gene, although disruption of such domains can also result in early embryonic mortality and so not be considered pathogenic. Calculation of *SDHA* Ka/Ks ratios for human versus marmoset (*Callithrix jacchus, calJac3 assembly)* and bushbaby *(Otelemur garnetti otoGar3 assembly)* revealed evidence for domains of strong purifying selection (Ka/Ks < 0.1) across multiple spans of the gene (Fig. 3). A total of 29.4% (*n* = 5/17) of the analyzed *SDHA* missense variants mapped to domains of strong purifying selection and 46% (*n* = 11/24) missense variants identified in healthy controls (see Table S6) (frequency <0.01%) (from Exome Variant Server (http://evs.gs.washington.edu). These frequencies are not significantly different (chi squared = 0.21, *P *=* *0.88).

## Discussion

To date, germline mutation analysis of *SDHA* has not been widely adopted in clinical practice. In part this relates to the more recent (compared to *SDHB/C/D*) association of *SDHA* mutations with tumorigenesis (Pantaleo et al. 2011a, 2011b; Burnichon et al. 2012; Dwight et al. 2013a; Jiang et al. 2015), but also to technical challenges (molecular genetic analysis of *SDHA* is complicated by its four known pseudogenes, generated by complete or partial gene duplications (Rattenberry et al. 2013).

Alhough the SDHA/B/C/D subunits form a single complex, mutations in different genes are associated with relative differences in susceptibility to specific tumor types. Thus, whilst *SDHA* is often not tested routinely for in PCC/PGL/HNPGL cases, it does appear to be a much rarer cause of PCC/PGL/HNPGL than *SDHB* and *SDHD* mutations. However, though other mutations in other SDH subunit genes may also be associated with GIST, the relative frequency of *SDHA* mutations reported in association with GIST appears much higher than other subunits (Boikos et al. 2016). Interestingly, *SDHA*‐associated GIST, has been reported to occur at an older age and have less female preponderance (Miettinen and Lasota 2014). Nevertheless, the tumorigenic effects of *SDHA* mutations are thought to be mediated through similar mechanisms as for mutations in other SDH subunits e.g. through a pseudohypoxic drive, facilitating angiogenesis and aberrant cell proliferation (López‐Jiménez et al. 2010) and epigenetic effects through the accumulation of succinate, and subsequent inhibition of demethylase enzymes resulting in promoter hypermethylation and tumor suppressor gene inactivation (Letouzé et al. 2013).

Since *SDHA* mutations were initially associated with PCC/PGL the spectrum of associated tumors has expanded to also include HNPGL, GIST, renal tumors, and pituitary adenoma (PA) (Pantaleo et al. 2011a, 2011b; Burnichon et al. 2012; Dwight et al. 2013a; Jiang et al. 2015). Thus the detection of a rare putative *SDHA* mutation might have clinical significance. However, *SDHA* mutations appear to have reduced penetrance (multiple affected individuals within a single family are rare) and *SDHA* mutations (e.g. c.91C>T p.Arg31*) can occur in healthy individuals at a population frequency of between 1/1000 and 1/10,000 (see Table 2). Thus the interpretation of the contribution of a putative novel germline *SDHA* mutation to the observed phenotype may not be straightforward as familial segregation studies are unlikely to be informative and the presence of the variant in control populations does not exclude pathogenicity. Interestingly a high variant density has been identified for *SDHA* in African American samples (Baysal et al. 2000). This increased variant expression was initially attributed to higher rates of gene recombination, however a study using a high resolution recombination map have disputed this theory as a low recombination rate at the locus of the *SDHA* gene was observed (Myers et al. 2005). It is now considered more likely that the four known *SDHA* pseudogenes have contributed to increased *SDHA* variant density by illegitimate recombination or gene conversion at the time of meiosis.

Bioinformatic prediction tools such as Polyphen and SIFT are widely used to aid the interpretation of the likely pathogenicity of sequence variants, although it is well recognized that they have their limitations. Previously, we and others have found that in silico structural prediction analysis tools can aid the classification of germline *SDHB* and *SDHD* variants (Ricketts et al. 2010). Although we found that most putative *SDHA* mutations detected in patients presenting with a relevant tumor were reported to impair protein stability, we also found that many rare *SDHA* missense variants present in the ESP6500 exome sequencing data set were also predicted to be destabilizing by DUET and pathogenic by PolyPhen/SIFT. Though no information is available on the phenotype of ESP6500 individuals with *SDHA* variants, this comparison does illustrate the challenge in interpreting the significance of rare genetic variants in candidate genes.

Identification of rare genetic variants associated with inherited tumor predisposition can enable testing of at risk relatives (and appropriate surveillance of mutation carriers), enhanced surveillance (if they are at increased risk of second primary tumors) and, if applicable, targeted therapy for the affected individual. In the case of putative *SDHA* mutations, the evidence for incomplete penetrance and lack of information on tumor risks in non‐probands suggests that the genetic testing and intensive surveillance of at risk family members will generally not be indicated until more information on the genetic epidemiology and age‐related tumor risks are available. For affected individuals with putative missense mutations, we suggest that, in addition to in silico protein structure and bioinformatic predictions of pathogenicity (e.g. SIFT/PolyPhen), additional studies should be undertaken to aid variant classification. Loss of heterozygosity (LOH) analysis of tumors can support a case for pathogenicity if there is loss of the wild type allele. Though the absence of LOH does not exclude pathogenicity of the variant as other mechanisms such as somatic point mutations or promoter hypermethylation can inactivate the wild‐type allele without causing LOH (as seen in our case with a c.91 C>T (p.Arg31*) variant and reported by others (Lussey‐Lepoutre et al. 2015). Tumor immunohistochemistry (IHC) can also support pathogenicity by demonstrating the loss of SDHA expression (Miettinen et al. 2013). However discrepancies between IHC results and predicted pathogenicity of *SDH* gene variants, appear to be more common for *SDHA* variants identified in patients with GIST (Evenepoel et al. 2015).

Furthermore, we suggest that there should also be an increased emphasis on defining whether a rare germline *SDHA* variant is associated with the expected functional consequences of SDHA inactivation in the relevant tumor. Thus metabolomic analysis using in vivo MRI spectroscopy (MRS) or in vitro high resolution magic angle spinning (HRMAS), have recently been reported as useful diagnostic adjunct in patients with putative SDHX gene mutations. Peaks in the metabolite succinate in tumor tissue as a result of a defective succinate dehydrogenase enzyme, have been demonstrated as a sensitive and specific hallmark of SDH mutations (Imperiale et al. 2015; Lussey‐Lepoutre et al. 2015) and have been described in an abdominal PGL associated with a germline *SDHA* c.91C>T (p.Arg31*) mutation (Lussey‐Lepoutre et al. 2015). Similarly, methylome profiling can be used to identify the hypermethylation epigenetic alterations associated with SDHx inactivation (Letouzé et al. 2013). The correct classification of putative *SDHA* mutations and the demonstration of the expected abnormal tumor metabolic/epigenetic profile will become increasingly important as targeted therapies based on derangements in the metabolic/epigenetic abnormalities are developed and studied.

In conclusion, this review of published *SDHA* mutations and reporting of variants from our novel cohort, should aid interpretation of genetic testing results in patients with relevant tumor types. We advise that caution should be exercised in interpreting pathogenicity of novel rare sequence variants and that, in such cases, whenever possible a variety of strategies, including structural prediction analysis and molecular genetics, SDHB/SDHA immunohistochemical analysis, metabolomic and methylome profiling of tumors should be performed, to better define the likelihood of pathogenicity of *SDHA* variants to ensure optimum clinical management.

## Conflict of Interest

None of the authors have anything to declare. This research has not been presented at any meeting to date.

## Supporting information


**Appendix S1.** Methods and Results.
**Table S1.** *SDHA* variants (both germline and somatic) described in literature.
**Table S2.** Characteristics of pathogenic variants reported in the literature.
**Table S3.** Variants associated with optic atrophy or Leigh syndrome.
**Table S4.** *SDHA* variants identified from EVS not associated with disease in our cohort or Literature.
**Table S5.** *SDHA* variants identified as somatic mutations in related tumor types in CBioportal.
**Table S6.** Variants that mapped to domains of strong purifying selection on mammalian alignment.
**Table S7.** Predicted effects of mutations on splicing. To determine the effect of exonic mutations on splicing we considered both whether they were at splice sites (distance = 0 – light green highlight) and whether they were predicted to have a significant effect on the density of exonic splice enhancers and suppressors. The change in exonic splice regulation score is given in column 3, with a Z score and P value (from simulation) in columns 4 and 5. A negative Z score is considered as a prediction of disrupted splicing. Mutations predicted to disrupt exonic splice enhancer motifs at *P* < 0.05 are shown in yellow.
