# Supplementary material for: SDHA related tumorigenesis: a new case series and literature review for variant interpretation and pathogenicity
Source: Mol Genet Genomic Med. 2017 Mar 2;5(3):237–50. doi: 10.1002/mgg3.279 (PMC5441402; doi:10.1002/mgg3.279)
Supplement: Supplementary file 1 — Appendix S1. Methods and Results. Table S1. SDHA variants (both germline and somatic) described in literature. Table S2. Characteristics of pathogenic variants reported in the literature. Table S3. Variants associated with optic atrophy or Leigh syndrome. Table S4. SDHA variants identified from EVS not associated with disease in our cohort or Literature. Table S5. SDHA variants identified as somatic mutations in related tumor types in CBioportal. Table S6. Variants that mapped to domains of strong purifying selection on mammalian alignment. Table S7. Predicted effects of mutations on splicing. To determine the effect of exonic mutations on splicing we considered both whether they were at splice sites (distance = 0 – light green highlight) and whether they were predicted to have a significant effect on the density of exonic splice enhancers and suppressors. The change in exonic splice regulation score is given in column 3, with a Z score and P value (from simulation) in columns 4 and 5. A negative Z score is considered as a prediction of disrupted splicing. Mutations predicted to disrupt exonic splice enhancer motifs at P < 0.05 are shown in yellow. [file MGG3-5-237-s001.docx]

**(Tables 1-3 in text and figures 1-3)**

**Supplementary data:**

**Methods including:**

- Predicting the effects of the variants on splice regulatory information
- Modelling of mammalian alignment to detect domains of purifying selection using *SDHA* transcript:

**Results including:**

- Predicting the effects of the variants on splice regulatory information

**Tables S1-S7**

- Table S1: *SDHA* variants (both germline and somatic) described in literature
- Table S2: Characteristics of pathogenic variants reported in the literature
- Table S3: Variants associated with Optic atrophy or Leigh syndrome.
- Table S4: *SDHA* variants identified from EVS not associated with disease in our cohort or Literature
- Table S5: *SDHA* variants identified as somatic mutations in related tumour types in CBioportal
- Table S6: Variants that mapped to domains of strong purifying selection on mammalian alignment.
- Table S7: Predicted effects of mutations on splicing. To determine the effect of exonic mutations on splicing we considered both whether they were at splice sites (distance = 0 - light green highlight) and whether they were predicted to have a significant effect on the density of exonic splice enhancers and suppressors. The change in exonic splice regulation score is given in column 3, with a Z score and P value (from simulation) in columns 4 and 5.  A negative Z score is considered as a prediction of disrupted splicing. Mutations predicted to disrupt exonic splice enhancer motifs at P<0.05 are shown in yellow

**Methods:**

**Predicting the effects of the variants on splice regulatory information**

To determine the possible impact of variants on splice forms we implemented the method of Di Giacomo et al (Di Giacomo et al., 2013), which has been shown to have good predictive power (Soukarieh et al., 2016). In brief this method considers the impact of each variant on the exonic splice enhancer (ESE) content of an exon. As ESEs typically function up to ~70bp from an exon end, we also calculate the distance to the nearest exon end to provide further confidence.

The ESRSeq scores of all exonic splice enhancers (ESEs, positive scores) and exonic splice silencers (ESSs, negative scores) were retrieved from Supplemental Table 4 in the paper by Ke et al (Ke et al., 2011), this being the source of splice motifs employed previously (Soukarieh et al., 2016). For all exonic variants, we calculated the mean of the ESRSeq scores of all ESEs and ESSs that overlapped with the variant position (based on the NM_004168.2 sequence retrieved as described above). Positions that overlapped with no putative ESEs or ESSs were scored 0. We then subtracted the mean score obtained with the reference sequence from that obtained after introducing the variant. A negative difference means that the variant is expected to increase levels of exon skipping while a positive difference indicates that an increase in exon inclusion is expected. For indels, the ESEs and ESSs that overlapped the base directly 5’ of the indel were considered. For deletions that affected both an exon and an intron, only the exonic portion was taken into account.

In order to determine the significance of the difference, we introduced the same variant at all other positions where the base present at the variant position occurred in the reference *SDHA* CDS (using the base immediately 5’ of the indel in the case of indels) and similarly calculated the change in average ESRSeq scores caused by the mutation. From this empirical distribution, we calculated a *p*-value ($p =\frac{n+1}{m+1}$, where *n* is the number of simulants presenting a difference as negative or more negative than the true variant and *m* is the total number of simulant positions analyzed) and a Z-score ($Z =\frac{true difference - mean of simulated differences}{standard deviation of simulated differences}$) for each variant.

For all exonic variants, we calculated the distance to the nearest splice junction based on the annotated gene structure of NM_004168.2 (the first and the last base of an exon were scored as having a distance of 0). For indels, the base immediately 5’ of the indel was considered as the variant position. All of the analysis described in this section was performed using custom Python 3.4.2. (www.python.org) scripts (standard library and NumPy) (Van der Welt et al., 2011)

**Modelling of mammalian alignment to detect domains of purifying selection using *SDHA* transcript:**

Mammalian alignment from the 100 vertebrate genomes alignment for NM_004168.2 was downloaded via Table Browser at UCSC <https://genome.ucsc.edu/cgi-bin/hgTables>. We calculated mean Ks (human to comparator) and % gap in the alignment and selected sequences with less than 5% gap and less than 0.45 Ks (to avoid saturation problem) but greater than 0.1 Ks (to ensure adequate information). Baboon sequence was eliminated owing to an in-frame stop. These filters resulted in two human-primate comparators, these being Human-marmoset (*Callithrix jacchus, calJac3 assembly)* and human- bushbaby *(Otelemur garnetti otoGar3 assembly).* The two alignments were passed by package SLIDERKK.tcl (available form LDH) to calculate Ka/Ks ratios in 108bp windows with overlapping windows and jumps of one codon between windows. We employed Li93 as the method of Ka/Ks calculation. We reviewed the variants identified in this study to determine which variants plotted to domains of strong purifying selection on this mammalian alignment.

**Results:**

**Prediction of splice disruption**

Experimental evidence suggests that between 30% and 77% of single nucleotide point mutations disrupt splicing. (Julien et al., 2016, Soukarieh et al., 2016, Pagani et al., 2005). Moreover, splice disrupting mutations tend to be at exon ends (Woolfe et al., 2010), and in the ClinVar database, there is a strong excess of pathogenic mutations both at splice sites, but more generally at exon ends (within ~70bp of a splice junction) (Wu et al., 2016). From this excess it is estimated that 25-45% of all disease causing mutations disrupt splicing (Wu et al., 2016), much in line with the experimentally observed figures. Mutations of any form (synonymous, missense and non-sense) can disrupt splicing, but here we don’t consider splicing mutations.

We first identified two exonic mutations that act at splice sites (c.457delC and c.1794G>C). In addition, we consider the effect of each mutation on exonic splice enhancer and exonic splice suppressor content. From this we identify four potentially pathogenic mutations that are predicted to significantly disrupt splicing. Note that this methodology has demonstrable accuracy (Soukarieh et al., 2016). Of the four mutations, three were previously characterized in the literature (c.553C>T, c.562C>T, c.1471G>T) and one is new to our cohort (c.1468G>T). In addition one EVS variant was identified (c.155C>T).

Of the six mutations seen in patients, all are likely to be pathogenic whether they disrupt splicing or not. The mutation c.562C>T and the splice site mutation c. 1794G>C are also both predicted to be pathogenic owing to protein disruption, assuming splicing is unaffected (Table 2). The variants; c.1468G>T, c.553C>T and c.1471G>T, all introduce a new stop codon and so are also likely to be pathogenic regardless of the effect on splicing. Likewise c.457delC both introduces a frameshift and is at a splice site, so is highly likely to be disruptive and pathogenic. The commonality of stop codon generating mutations in the predicted splice-disrupting list probably relates to the paucity of stop codons in exonic splice motifs and to the fact that some fraction of the time the motifs appear in frame. More generally, ESEs have a very low T content (Caceres et al., 2013), while stop codons have a high T content.

**Table S1**

| **Nucleotide** | **Protein change** | **Effect on protein** | **Affected individuals** | **Germline** | **Mean age** | **References** |
| --- | --- | --- | --- | --- | --- | --- |
| c.2T>C | (p.Met1Thr) | Translation | 1 GIST,1 RCC | Yes | 23 | (Jiang et al.2015) |
| c.91C>T | (p.Arg31*) | Truncating | 15 GIST, 5PGL, 2 HNPGL,1 PA | Yes | 33 | (Burnichon et al. 2010)  (Belinsky et al, 2013a)  (Italiano et al. 2012)  (Oudijk et al. 2013)  (Pantaleo et al. 2013)  (Pantaleo et al. 2011b)  (Niemeijer et al. 2015)  (Wagner et al. 2013) |
| c.113A>T | (p.Asp38Val) | Missense | GIST | No | 26 | (Italiano et al. 2012) |
| c.160C>T+  c.800 C>T ^*^  * = (somatic second hit) | (p.Gln54*)  (p.Thr267Met) | Missense | GIST | Yes | 29 | (Dwight et al. 2013b) |
| c.457-2_c457delAGC | (p.Leul153Lysfs*71) | Truncating | GIST | Yes | 52 | (Belinsky et al. 2013a) |
| c.457-3_457-1 delCAG | N/A | Splice site | 1 GIST | Yes | 37 | (Pantaleo et al. 2013)  (Belinsky et al. 2013b) |
| c.511C>T | (p.Arg171Cys) | Missense | 1 GIST | No | 26 | (Pantaleo et al. 2013) |
| c.553C>T | (p.Gln185*) | Missense | GIST | Yes | 31 | (Wagner et al. 2013) |
| c.562C>T | (p.Arg188Trp) | Missense | GIST | Yes | N/A | (Miettinen et al. 2013)  (Belinsky et al. 2013b) |
| c.622-2_622-2delA | N/A | Splice site | RCC | No | 62 | (Ozluk et al. 2015) |
| 17k bp SDHA homozygous deletion on chrm 5p.15 | N/A | Truncating | RCC | No | 54 | (Yakirevich et al. 2015) |
| c.725_736del+ c.989_990insTA | N/A | Truncating | 1 PA | No | 62 | (Gill et al. 2014) |
| c.767C>T | (p.Thr256Ile) | Missense | 1 GIST | Yes | 34 | (Miettinen et al. 2013) |
| c.818C>T | (p. Thr273Ile) | Missense | 2 GIST | Yes | 21 | (Miettinen et al. 2013)  (Belinsky et al. 2013b) |
| c.1046_1047delTG | (p.Leu349Arg fs*11) | Truncating | 1 GIST | No | 18 | (Papathomas et al. 2015) |
| c.1151C>G | (p.Ser384*) | Truncating | 1 GIST | Yes | 28 | (Pantaleo et al. 2013) |
| c.1255G>A | (p.Gly419Arg) | Missense | 1 GIST | No | 17 | (Pantaleo et al. 2013 |
| c.1334C>T | (p.Ser445Leu) | Missense | 1 GIST | No | 34 | (Italiano et al. 2012) |
| c.1361 C>A | (p.Ala454Glu) | Missense | 1 GIST | No | 21 | (Italiano et al. 2012) |
| c.1471G>T | (p.Glu491*) | Missense | 1 GIST | No | 48 | (Belinsky et al. 2013a) |
| c.1534C>T | (p.Arg512*) | Truncating | 1HNPGL,1 GIST | Yes | 23 | (Wagner et al. 2013 |
| c.1690G>A | (p.Glu564Lys) | Missense | 1 GIST | No | 17 | (Pantaleo et al. 2013) |
| c.1753C>T | (p.Arg585Trp) | Missense | 1 PC, 1 PGL | Yes | 25 | (Korpershoek et al. 2011) |
| c.1765C>T | (p.Arg589Trp) | Missense | 2 GIST,2 PGL | Yes | 31 | (Burnichon et al. 2010)  (Wagner et al. 2013) |
| c.1766 G>A | (p.Arg589Gln) | Missense | 1 GIST | Yes | 38 | (Pantaleo et al. 2013) |
| c.1794G>C | (p.Lys598Asn) | Missense | 1 GIST | Yes | 34 | (Miettinen et al. 2013) |
| c.1795-1G>T | N/A splicing | Splice site | 1 GIST | Yes | 34 | (Miettinen et al. 2013) |
| c.1873C>T | (p.His625Tyr) | Missense | 1 PA, 1 HNPGL | Yes | 38 | (Dwight et al. 2013b) |

**Table S2**

| **Nucleotide** | **SDHA Protein Expression by IHC** | **LOH in tumour** | **SIFT** | **Polyphen** | **Frequency of allele per 1000 healthy population** |
| --- | --- | --- | --- | --- | --- |
| c.91C>T | Lost in 17/17 tumours | LOH in 13/17 tumours | Damaging | Damaging | 0.16 |
| c.457-2_c457delAGC | Lost1/1 | LOH | N/A | N/A | Not described |
| c.1046_1047delTG | Lost1/1 | LOH 1/1 | Damaging | Damaging | Not described |
| c.1151C>G | Lost 1/1 | LOH in 1/1 | Damaging | Damaging | 0.005 |
| c.1534C>T | Lost2/2 | LOH 2/2 | Benign | Benign | Not described |
| c.2T>C | Lost1/1 | Homozygous in both tumour types | N/A | N/A | .016 |
| c.457-3_457-1 | Lost1/1 | Homozygous in tumour | Damaging | Damaging | Not described |
| c.553 C>T | Lost1/1 | Second hit- 13-bp deletion | Damaging | Probably damaging | .016 |
| c.725_736del+ c.989_990insTA | Lost in 1/1 | 2 somatic mutations | N/A | N/A | Not described |
| c.622-2_622-2delA | Lost in 1/1 | Homozygous in tumour | N/A | N/A | Not described |
| 17Kbp SDHA homozygous deletion on chrm 5p15 | Lost1/1 | Homozygous in tumour | N/A | N/A | Not described |
| c.113A>T | Lost1/1 | LOH 1/1 | Benign | Benign | 35.0 |
| c.160C>T | Lost 1/1 | Second somatic mutation | Damaging | Probably damaging | Not described |
| c.511C>T | Lost 1/1 | Second somatic mutation | Damaging | Damaging | Not described |
| c.562C>T | Retained1/1 | LOH 1/1 | N/A | N/A | Not described |
| c.767C>T | Lost in 1/1 | LOH in 1/1 | Damaging | Probably damaging | Not described |
| c.800C>T | Lost 1/1 | LOH 1/1 | Damaging | Probably damaging | .025 |
| c.1255G>A | Not performed | Two somatic mutations | Damaging | Probably damaging | Not described |
| c.1334C>T | Lost in 1/1 | LOH in 1/1 | Damaging | Probably damaging | Not described |
| c.1471G>T | Not performed | LOH | Benign | Possibly damaging | Not described |
| c.1690G>A | Not performed | Second somatic mutation | Damaging | Probably damaging | Not described |
| c.1753C>T | Lost in 1/2 | LOH in 2/2 | Benign | Benign | 0.025 |
| c.1765C>T | Lost4/4 | LOH 4/4 | Benign | Benign | Not described |
| c.1766G>A | Lost 1/1 | Second somatic mutation | Benign | Probably damaging | Not described |
| c.1794G>C | Lost in 1/1 | LOH in 1/1 | N/A | N/A | Not described |
| c.1795-1G>T | Lost in 1/1 | LOH in 1/1 | N/A | N/A | Not described |
| c.1873C>T | Lost2/2 | Retention of WT allele | N/A | N/A | Not described |

**Table S3.**

| **Nucleotide** | **Protein change** |
| --- | --- |
| c.1660C>T | (p.Arg554Trp) |
| c.1571C>T | (p.Ala524Val) |
| c.356G>A | (p.Trp119*) |
| c.248C>T | (p.Ala83Val) |
| c.91C>T | (p.Arg31*) |
| c.1A>C | (p.Met1Val) |
| c.64-2A>G | N/A |
| c.1065-3C>A | N/A |
| c.1664G>A | (p.Gly555Glu) |

**Table S4**

| **Variant** | **Frequency per 1000** | **DUET score** | **mCSM-PPI score** | **Effect on protein** | **SIFT and Polyphen prediction** |
| --- | --- | --- | --- | --- | --- |
| c.276G>C  (p.Lys92Asn) | .008 | -0.766 | -1.112 | Destabilses protomer and complex and substrate binding site | Probably damaging |
| c.287C>T  (p.Thr96Ile) | .0082 | 0.341 | -0.649 | Destabilises complex | Probably damaging |
| c.607A>G)  (p.Thr203Ala) | .041 | -1.191 | -0.144 | Destabilises protomer | Probably damaging |
| c.704T>C  (p.Ile235Thr) | .082 | -2.713 | 0 | Destabilises protomer | Possibly damaging |
| c.155C>T  (p.Ser52Phe) | .12 | -0.881 | 0 | Destabilises protomer | Possibly damaging |
| c.830C>T  (p.Thr277Met) | .10 | 0.19 | 0 | No effect | Probably damaging |
| c.861C>G  (p.Cys287Trp) | .0082 | -1.703 | 0 | Destabilises protomer | Probably damaging |
| c.986 G>A  (p.Arg329Gln) | .016 | -0.466 | 0 | Mildly destabilises protomer | Probably damaging |
| c.1042A>T  (p.Thr348Ser) | .0082 | -1.219 | 0 | Destabilses protomer | Probably damaging |
| c.1055G>A  (p.Arg352Gln) | .065 | -0.228 | 0 | No effect | Probably damaging |
| c.1090 G>A  (p.Val364Ile) | .024 | -0.975 | 0 | Destabilises protomer | Benign |
| c.1122 G>T  (p.Glu374Asp) | .0082 | 0.052 | -0.471 | Destabilises complex | Benign |
| c.1171 G>A  (p.Ala391Thr) | .024 | -0.736 | -0.007 | Destabilises protomer | Probably damaging |
| c.1352 G>A  (p.Arg451His) | .0082 | -1.939 | -0.774 | Destabilises protomer, complex and cofactor binding | Probably damaging |
| c.1430 C>T  (p.Pro477Leu) | .016 | -0.393 | -0.725 | Destabilises complex | Benign |
| c.1492 A>C  (p.Lys498Gln) | .0083 | -0.189 | -0.535 | Destabilises complex | Benign |
| c. 1532T>C  (p.Leu511Pro) | .050 | -2.113 | -1.079 | Destabilises protomer and complex | Possibly damaging |
| c. 1597 C>G  (p.Gln533Glu) | .024 | 0.073 | 0 | No effect | Benign |
| c.1751 C>T  (p.Ala584Val) | .14 | -0.285 | 0 | No effect | Probably damaging |
| c.1772 C>T  (p.Ala591Val) | .008 | -0.55 | 0 | Mildly destabilises protomer | Probably damaging |
| c.1786G>T  (p.Asn596Tyr) | .008 | -0.597 | 0 | Mildly destabilises protomer | Probably damaging |
| c.1951G>A  (p.Glu651Lys) | .032 | 0.492 | 0 | No effect | Possibly damaging |
| c.1973C>T  (p.Pro658Leu) | .082 | -0.234 | 0 | No effect | Probably damaging |
| c.1979 C>G  (p.Ala660Gly) | .11 | -0.793 | 0 | Destabilises protomer | Probably damaging |

**Table S5.**

| **Variant** | **Tumour type** | **Duet score** | **mCSM-PPI score** | **Effect on protein** | **SIFT and polyphen prediction** |
| --- | --- | --- | --- | --- | --- |
| * (p.Met388Ile) | RCC | -0.083 | -1.001 | Destabilises complex | Possibly damaging |
| c.1367C>T (p.Ser456Leu) | RCC | 0.66 | 0 | Destabilises co factor binding and substrate binding site | Probably damaging |
| c.1396G>A (p.Ala466Thr) | RCC | -1.671 | 0 | Destabilises protomer, | Probably damaging |
| c.1360G>T  (p.Ala454Thr) | RCC | -2.212 | -0.955 | Destabilises protomer, complex, co factor binding and substrate binding site | Probably damaging |
| c.2044C>T  (p.Pro643Ala) | RCC | -1.416 | 0 | Destabilises protomer | Probably damaging |
| * (p.Ile383Phe) | RCC | -1.924 | -1.182 | Destabilises protomer and complex formation | Damaging |
| c.17G>A (p.Gly6Asp) | RCC | --------- | --------- | Transit peptide | Benign |
| c.2074C>T (p.Asp653Ala) | PC | -0.69 | 0 | Destabilises protomer | Probably damaging |

***Nucleotide sequence not reported on cBioportal (www.cBioprtal.org) or Ensembl (www.ensembl.org)**

**Table S6.**

| Variant | Variant source |
| --- | --- |
| c.276G>C (p.Lys92Asn) | EVS |
| c.287C>T (p.Thr96Ile) | EVS |
| c.511C>T (p.Arg171Cys) | Literature review |
| c.562C>T (p.Arg188Trp) | Literature review |
| c.704T>C (p.Ile235Thr) | EVS |
| c.923C>T (p.Thr308Met) | UK cohort |
| c.986 G>A (p.Arg329Gln) | EVS |
| c.1042A>T (p.Thr348Ser) | EVS |
| c.1055G>A (p.Arg352Gln) | EVS |
| c.1090 G>A (p.Val364Ile) | EVS |
| c.1523C>T (p.Thr508Ile) | EVS |
| c. 1532T>C (p.Leu511Pro) | EVS |
| c.1786C>T (p.Asp596Tyr) | EVS |
| c.1794G>C (p.Lys598Asn) | Literature Review |
| c.1873 C>T (p.His625Tyr) | Literature Review |
| c.1913C>T (p.Thr638Ile) | EVS |

**Table S7.**

| Position | Mutation | Change in average ESRseq score | Z-score for change | Empirical one tailed P for change | Distance to nearest splice junction | Number of simulants | Variant source |
| --- | --- | --- | --- | --- | --- | --- | --- |
| 2 | c.2T>C | 0.4725 | 1.026860761 | 0.844339623 | 61 | 423 | literature |
| 68 | c.68C>T | -0.283 | -0.344664385 | 0.367265469 | 4 | 500 | EVS |
| 91 | c.91C>T | 0.1455 | 1.1890866 | 0.878243513 | 27 | 500 | novel UK cohort + literature |
| 113 | c.113A>T | -0.2993333 | -0.462448087 | 0.269565217 | 37 | 459 | literature |
| 133 | c.133G>A | -0.5625 | -1.264589649 | 0.124590164 | 17 | 609 | novel UK cohort |
| 136 | c.136A>G | 0.69008333 | 1.725499466 | 0.965217391 | 14 | 459 | novel UK cohort |
| 155 | c.155C>T | -0.6571667 | -1.687178015 | 0.03992016 | 4 | 500 | EVS |
| 160 | c.160C>T | -0.4952 | -1.104655679 | 0.145708583 | 9 | 500 | literature |
| 276 | c.276G>C | 0.1 | 0.154300548 | 0.567213115 | 36 | 609 | EVS |
| 287 | c.287C>T | -0.1541667 | 0.11600082 | 0.526946108 | 25 | 500 | EVS |
| 457 | c.457delC | -0.1803333 | -0.39777037 | 0.30738523 | 0 | 500 | literature |
| 511 | c.511C>T | -0.477 | -1.03935999 | 0.165668663 | 54 | 500 | literature |
| 553 | c.553C>T | -0.69175 | -1.811962779 | 0.025948104 | 68 | 500 | literature |
| 562 | c.562C>T | -0.669 | -1.729857238 | 0.033932136 | 59 | 500 | literature |
| 607 | c.607A>G | 0.21133333 | 0.485299809 | 0.652173913 | 14 | 459 | EVS |
| 704 | c.704T>C | 0.1915 | 0.011640857 | 0.530660377 | 66 | 423 | EVS |
| 725 | c.725delGGTCCATCCATC | -0.1106667 | -0.212526543 | 0.393442623 | 45 | 609 | literature |
| 767 | c.767C>T | -0.1611333 | 0.091091827 | 0.512974052 | 3 | 500 | literature |
| 800 | c.800C>T | -0.41225 | -0.807261437 | 0.229540918 | 29 | 500 | literature |
| 818 | c.818C>T | -0.2365 | -0.178375908 | 0.421157685 | 47 | 500 | literature |
| 830 | c.830C>T | -0.1375 | 0.175593639 | 0.582834331 | 59 | 500 | EVS |
| 861 | c.861C>G | 0.27916667 | 0.761302955 | 0.760479042 | 34 | 500 | EVS |
| 923 | c.923C>T | -0.5386667 | -1.260717225 | 0.111776447 | 27 | 500 | novel UK cohort |
| 986 | c.986G>A | -0.3961667 | -0.839975779 | 0.231147541 | 78 | 609 | EVS |
| 989 | c.989_990insTA | 0.002 | 1.015592253 | 0.876086957 | 75 | 459 | literature |
| 1003 | c.1003A>G | 0.0333333 | 0.02609158 | 0.508695652 | 61 | 459 | EVS |
| 1042 | c.1042A>T | -0.1386 | 0.000273528 | 0.441304348 | 22 | 459 | EVS |
| 1046 | c.1046_1047delTG | 0.0810667 | 0.066347961 | 0.535377358 | 18 | 423 | literature |
| 1055 | c.1055G>A | 0.3116667 | 0.963209287 | 0.816393443 | 9 | 609 | EVS |
| 1090 | c.1090G>A | -0.393 | -0.831900361 | 0.23442623 | 25 | 609 | EVS |
| 1122 | c.1122G>T | -0.019 | 0.379192549 | 0.596721311 | 57 | 609 | EVS |
| 1151 | c.1151C>G | -0.09 | -0.21316302 | 0.399201597 | 86 | 500 | literature + EVS |
| 1171 | c.1171G>A | -0.13475 | -0.17399271 | 0.419672131 | 89 | 609 | EVS |
| 1255 | c.1255G>A | 0.3525833 | 1.067637733 | 0.847540984 | 5 | 609 | literature |
| 1273 | c.1273G>A | -0.2025 | -0.346497035 | 0.380327869 | 12 | 609 | novel UK cohort |
| 1334 | c.1334C>T | -0.544167 | -1.280476902 | 0.105788423 | 73 | 500 | literature |
| 1338 | c.1338delA | 0.5355 | 1.817889658 | 0.954347826 | 77 | 459 | novel UK cohort |
| 1352 | c.1352G>A | -0.318 | -0.640712739 | 0.295081967 | 80 | 609 | EVS |
| 1361 | c.1361C>A | -0.0468 | 0.018593517 | 0.481037924 | 71 | 500 | literature + EVS |
| 1381 | c.1381G>T | -0.15625 | 0.015052619 | 0.460655738 | 51 | 609 | EVS |
| 1430 | c.1430C>T | -0.441333 | -0.911474928 | 0.209580838 | 2 | 500 | EVS |
| 1468 | c.1468G>T | -0.87925 | -1.908595909 | 0.031147541 | 35 | 609 | novel UK cohort |
| 1471 | c.1471G>T | -0.8552 | -1.844242212 | 0.037704918 | 38 | 609 | literature |
| 1492 | c.1492A>C | 0.2 | 0.425010477 | 0.7 | 59 | 459 | EVS |
| 1523 | c.1523C>T | -0.0526 | 0.479244451 | 0.694610778 | 28 | 500 | EVS |
| 1532 | c.1532T>C | 0.159 | -0.105631459 | 0.481132075 | 19 | 423 | EVS |
| 1534 | c.1534C>T | -0.15805 | 0.102116095 | 0.518962076 | 17 | 500 | literature |
| 1597 | c.1597C>G | -0.4521 | -1.170122799 | 0.127744511 | 45 | 500 | EVS |
| 1690 | c.1690G>A | 0.38305 | 1.145432342 | 0.875409836 | 26 | 609 | literature |
| 1696 | c.1696C>G | 0.1625833 | 0.453359858 | 0.678642715 | 32 | 500 | EVS |
| 1751 | c.1751C>T | -0.134 | 0.188108564 | 0.588822355 | 43 | 500 | EVS |
| 1753 | c.1753C>T | -0.0784 | 0.386948624 | 0.662674651 | 41 | 500 | novel UK cohort + literature |
| 1765 | c.1765C>T | -0.399333 | -0.760994753 | 0.241516966 | 29 | 500 | novel UK cohort + literature |
| 1766 | c.1766G>A | -0.4156 | -0.889539345 | 0.22295082 | 28 | 609 | literature |
| 1772 | c.1772C>T | -0.37225 | -0.664014944 | 0.265469062 | 22 | 500 | EVS |
| 1786 | c.1786G>T | -0.29575 | -0.355048075 | 0.337704918 | 8 | 609 | EVS |
| 1794 | c.1794G>C | 0.6118 | 1.679754831 | 0.952459016 | 0 | 609 | literature |
| 1873 | c.1873C>T | -0.1725 | 0.050451525 | 0.50499002 | 35 | 500 | literature |
| 1913 | c.1913C>T | 0.225 | 1.474845861 | 0.94011976 | 4 | 500 | EVS |
| 1951 | c.1951G>A | -0.135417 | -0.175690004 | 0.418032787 | 42 | 609 | EVS |
| 1973 | c.1973C>T | 0.21375 | 1.434367704 | 0.934131737 | 64 | 500 | EVS |
| 1979 | c.1979C>G | 0.1461667 | 0.410021615 | 0.658682635 | 70 | 500 | EVS |
